# Lung Transplant Recipient with Pulmonary Alveolar Proteinosis

**DOI:** 10.1155/2016/4628354

**Published:** 2016-04-24

**Authors:** Sofya Tokman, M. Frances Hahn, Hesham Abdelrazek, Tanmay S. Panchabhai, Vipul J. Patel, Rajat Walia, Ashraf Omar

**Affiliations:** ^1^Norton Thoracic Institute, St. Joseph's Hospital and Medical Center, Phoenix, AZ 85013, USA; ^2^Department of Pathology, St. Joseph's Hospital and Medical Center, Phoenix, AZ 85013, USA

## Abstract

Pulmonary alveolar proteinosis (PAP) is a progressive lung disease characterized by accumulated surfactant-like lipoproteinaceous material in the alveoli and distal bronchioles. This accumulation is the result of impaired clearance by alveolar macrophages. PAP has been described in 11 solid organ transplant recipients, 9 of whom were treated with mammalian target of rapamycin inhibitors. We report a case of a lung transplant recipient treated with prednisone, mycophenolate mofetil (MMF), and tacrolimus who ultimately developed PAP, which worsened when MMF was replaced with everolimus.

## 1. Introduction

Pulmonary alveolar proteinosis (PAP) is a progressive lung disease characterized by accumulation of surfactant-like lipoproteinaceous material within the alveoli and distal bronchioles due to impaired clearance by alveolar macrophages. Most patients have worsening exertional dyspnea and cough, while few have fever, chest pain, or hemoptysis in the absence of a superimposed infection. The serum level of lactate dehydrogenase is frequently elevated in patients with PAP, and their bronchoalveolar lavage (BAL) fluid has an opaque, milky appearance and is predominantly composed of macrophages and lymphocytes. High-resolution computed tomography (HRCT) reveals patchy, ground-glass opacities with interlobular septal thickening in a characteristic “crazy paving” pattern [1]. Open lung biopsy has historically been the gold standard for PAP diagnosis; however, up to 75% of cases can be diagnosed via BAL [1].

To date, 9 reports have described 11 solid organ recipients who developed PAP. Of these, 3 were lung transplant recipients [2, 3] and 8 were kidney transplant recipients [4–10]. Nine of these patients were on immunosuppressive regimens that included mammalian target of rapamycin- (mTOR-) inhibitors (8 were treated with sirolimus [2, 5–7, 9] and 1 was treated with everolimus [4]), while 2 were on mTOR-inhibitor sparing regimens that included prednisone, a calcineurin inhibitor, and mycophenolate mofetil (MMF) [3, 10]. Here we report our experience with a lung transplant recipient treated with prednisone, MMF, and tacrolimus who developed PAP that worsened when MMF was replaced with everolimus.

## 2. Case Presentation

The patient was a 67-year-old man who underwent bilateral lung transplantation for smoking-related chronic obstructive lung disease. His posttransplant course was complicated by mildly reduced left ventricular systolic function with an ejection fraction of 40%, impaired left ventricular relaxation with diastolic dysfunction, prostate cancer with radiation proctitis, and calcineurin inhibitor-induced renal insufficiency. He was maintained on a standard three-drug immunosuppressive regimen of prednisone, tacrolimus, and MMF for 35 months after transplant but was ultimately transitioned to a combination of prednisone, lower-dose tacrolimus, and everolimus to minimize the risk of prostate cancer recurrence and to slow the progression of calcineurin inhibitor-induced renal insufficiency.

**Figure 1:**
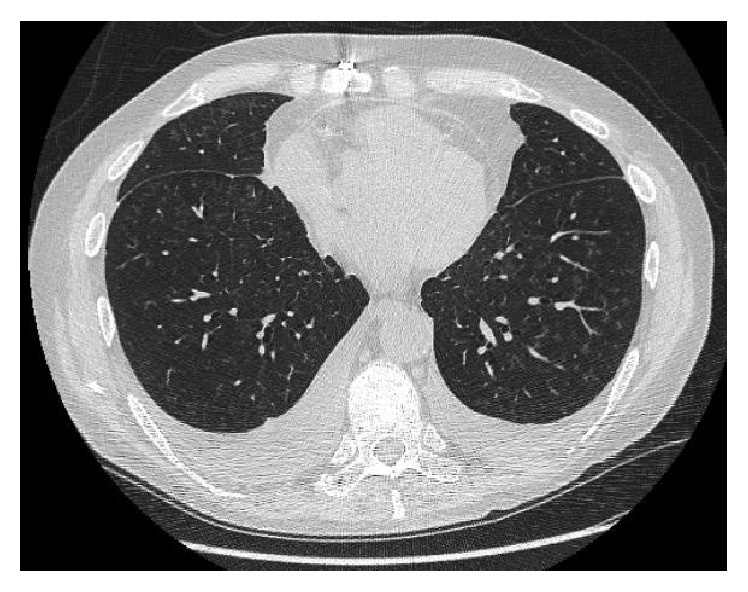
High-resolution axial computed tomography scan of the chest shows ground-glass centrilobular nodules before initiation of everolimus therapy.

**Figure 2:**
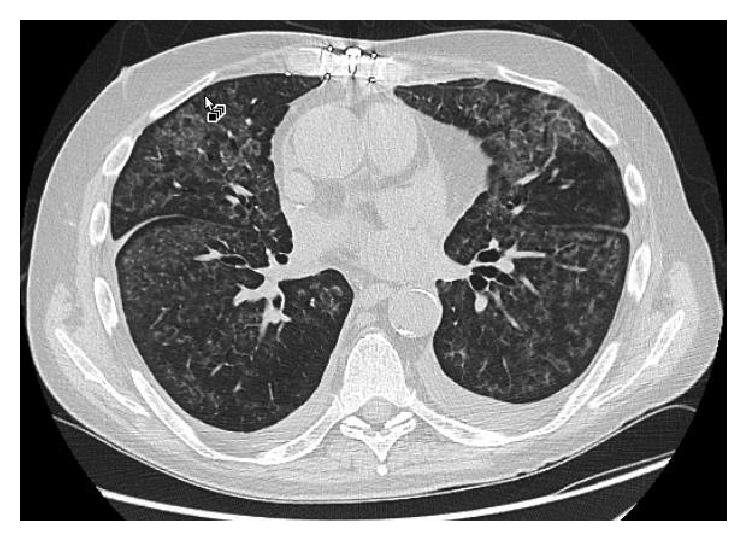
High-resolution axial computed tomography scan of the chest shows diffuse ground-glass abnormalities with interlobular septal thickening in the characteristic “crazy paving” pattern after initiation of everolimus therapy.

The patient experienced good allograft function for 3.5 years after transplant as evidenced by stable spirometry and absence of respiratory symptoms. Radiographically, he developed diffuse, centrilobular ground-glass nodules and small pleural effusions 25 months after transplant (Figure 1). The etiology of these nodules and effusions was unknown, despite multiple bronchoscopies with BAL fluid analysis and transbronchial biopsies, all of which showed normal lung parenchyma and no evidence of infection. At 41 months after lung transplant (16 months after onset of ground-glass nodules and 6 months after initiation of everolimus therapy), the patient returned to our clinic with worsening dyspnea; declines of 16% and 20% in FEV1 and FVC, respectively; and a “crazy paving” pattern on HRCT (Figure 2).

**Figure 3:**
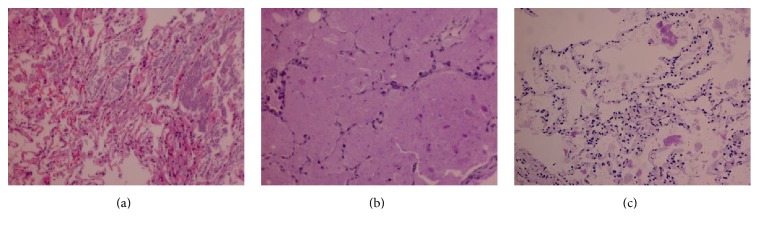
Samples obtained via video-assisted thoracoscopic biopsy after initiation of everolimus therapy. (a) Pink granular material filling the airspaces, hematoxylin and eosin stain. (b) Scattered amorphous solid eosinophilic globules, periodic acid-Schiff (PAS) stain. (c) Diastase-resistant eosinophilic globular inclusions, PAS stain with diastase.

**Figure 4:**
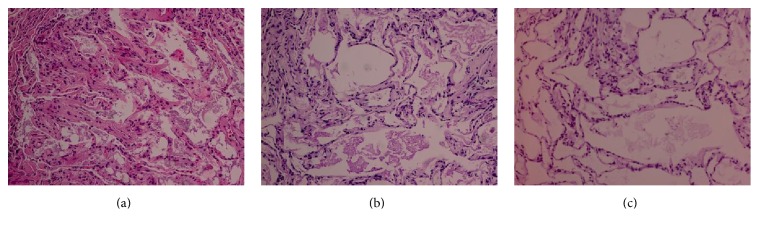
Samples obtained via transbronchial biopsy before initiation of everolimus therapy. (a) Pink granular material partially filling the airspaces, hematoxylin and eosin stain. (b) Eosinophilic material within airspaces, periodic acid–Schiff (PAS) stain. (c) Rare, small diastase-resistant globular inclusions, PAS stain with diastase.

The patient was hospitalized and underwent bronchoscopy with BAL, which returned milky fluid characteristic of PAP. A right middle lobe biopsy via video-assisted thoracic surgery revealed pink proteinaceous material filling the air spaces with diastase-resistant periodic acid-Schiff (PAS) stain-positive globular inclusions consistent with PAP (Figure 3). Cultures and special stains (i.e., gram stain and methenamine fungal stain) showed no evidence of infection. Everolimus therapy was halted and the patient was treated with granulocyte-macrophage colony stimulating factor (GM-CSF), with some symptomatic and radiographic improvement. Serum lactate dehydrogenase was not measured and although anti-GM-CSF antibody titers were sent for testing, the results were never available. The patient's hospital course was further complicated by serotonin syndrome with hemodynamic instability that prohibited whole-lung lavage (WLL), nonoliguric renal failure requiring hemodialysis, and sepsis due to acalculous cholecystitis that ultimately led to his death after a month-long hospitalization. Postmortem reanalysis of transbronchial biopsies obtained before initiation of everolimus therapy revealed scattered diastase-resistant PAS-positive globular inclusions within a background of pink granular material in 1 of 5 biopsies, consistent with PAP in the absence of mTOR-inhibitor therapy (Figure 4).

## 3. Discussion

Three clinically distinct forms of PAP have been identified: primary (90%), secondary (5–10%), and congenital (2%) [11]. Primary PAP is caused by development of autoantibodies to GM-CSF, the presence of which impairs clearance of surfactant by alveolar macrophages [12]. A PAP-like histopathology has been observed in GM-CSF knockout mice, which have been used to shed light on the mechanism of PAP development in humans. Alveolar macrophages isolated from these mice show decreased expression of PU.1, which is a transcription factor required for functional maturation of alveolar macrophages. When provided with GM-CSF in vitro, these immature macrophages expressed PU.1 and cell surface markers characteristic of mature macrophages and acquired the ability to metabolize surfactant [13].

Secondary PAP develops in association with conditions that lead to functional impairment or reduced numbers of alveolar macrophages, including some hematologic malignancies, certain infections, inhalation of inorganic dust or toxic fumes, and pharmacologic immunosuppression [1]. Our patient developed centrilobular ground-glass nodules before initiation of everolimus therapy, and these nodules progressed to a “crazy paving” pattern after everolimus was started. He may have suffered from immunosuppression-induced macrophage dysfunction and resultant mild PAP that worsened significantly with the addition of an mTOR-inhibitor.

Although the exact mechanism of mTOR-inhibitor-associated PAP is unclear, an association between this drug class and development of PAP likely exists, as our case is the seventh report of this complication in solid organ transplant recipients treated with mTOR-inhibitors. Of the 9 patients described in the literature, 8 were treated with sirolimus [2, 5–7, 9] and only 1 was treated with everolimus [4]. We speculate that the difference between the two drugs may be related to the more hydrophilic nature of everolimus [4].

Treatment of patients with drug-induced secondary PAP should focus first on removing the offending agent. Administration of GM-CSF is controversial, as antibodies against GM-CSF are absent [14]; however, in severe cases, it may be reasonable to give this drug while awaiting anti-GM-CSF serologic test results. WLL is a therapeutic option for patients with severe disease. It involves bronchoscopic instillation of 1 L aliquots of warmed normal saline via a dual lumen endotracheal tube until the milky effluent becomes clear [15]. Patients who are too hypoxemic to tolerate WLL under general anesthesia alone can be supported with venovenous or venoarterial extracorporeal membrane oxygenation [16]. Alternatively, WLL can be performed in a hyperbaric chamber [9].

The prognosis of solid organ transplant recipients with PAP remains largely unknown. Of the 11 patients identified in the literature, clinical outcomes were reported in 8. One patient continued treatment with everolimus and died of pneumonia and sepsis within 6 months of PAP diagnosis [4], 5 improved with cessation of sirolimus therapy (2 of whom also underwent WLL) [5–9], 1 improved with replacement of MMF and tacrolimus with cyclosporine [3], and another one improved with replacement of MMF and cyclosporine with azathioprine [10]. None of these patients were treated with GM-CSF. Although PAP remains an uncommon complication of immunosuppressive therapy, it must be considered in the appropriate clinical setting.
